# hESCs‐Derived Early Vascular Cell Spheroids for Cardiac Tissue Vascular Engineering and Myocardial Infarction Treatment

**DOI:** 10.1002/advs.202104299

**Published:** 2022-01-29

**Authors:** Yang Liu, Yifan Zhang, Tianxiao Mei, Hao Cao, Yihui Hu, Wenwen Jia, Jing Wang, Ziliang Zhang, Zhan Wang, Wenjun Le, Zhongmin Liu

**Affiliations:** ^1^ Institute for Regenerative Medicine Shanghai East Hospital Frontier Science Center for Stem Cell Research School of Medicine Tongji University Shanghai 200092 China; ^2^ National Stem Cell Translational Resource Center Shanghai East Hospital School of Life Sciences and Technology Tongji University Shanghai 200092 China; ^3^ Department of Cardiovascular Surgery Shanghai East Hospital Tongji University School of Medicine Shanghai 200120 China; ^4^ Department of Internal Medicine Section on Molecular Medicine Wake Forest School of Medicine Medical Center Blvd Winston‐Salem NC 27157 USA

**Keywords:** 3D bioprinting, cell spheroids, embryonic stem cells, myocardial infarction, vascularized tissues

## Abstract

Transplanting functional cells to treat myocardial infarction (MI), a major disease threatening human health, has become the focus of global therapy. However, the efficacy has not been well anticipated, partly due to the lack of microvascular system that supplies nutrients and oxygen. Here, spheroids of early vascular cells (EVCs) derived from human embryonic stem cells (hESCs), rather than single‐cell forms, as transplant “seeds” for reconstructing microvascular networks, are proposed. Firstly, EVCs containing CD34^+^ vascular progenitor cells are identified, which effectively differentiate into endothelial cells in situ and form vascular networks in extracellular matrix (ECM) hydrogel. Secondly, cardiac microtissues and cardiac patches with well‐organized microvasculature are fabricated by three‐dimensional (3D) co‐culture or bioprinting with EVCs and cardiomyocytes in hydrogel. Notably, in 3D‐bioprinted myocardial models, self‐assembly vascularization of EVC spheroids is found to be significantly superior to EVC single cells. EVC spheroids are also injected into ischemic region of MI mouse models to explore its therapeutic potential. These findings uncover hESCs‐derived EVC spheroids rather than single cells are more accessible for complex vasculature engineering, which is of great potential for cardiac tissue vascular engineering and MI treatment by cell therapy.

## Graphical abstract

Taking advantages of the in situ differentiation and self‐assembly vascularization of human embryonic stem cells (hESCs) derived early vascular cells (EVCs), spheroids of EVCs rather than single cells are identified as seeding cells for vascular engineering of 3D bioprinted cardiac patches and as an alternative cell delivery strategy for cell therapy of myocardial infarction.

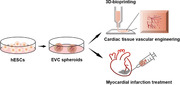

## 1 Introduction

Myocardial infarction (MI) induced myocardium ischemia can cause massive loss of cardiomyocytes (CMs) which have limited regenerative capacity in adult heart.^[^ ^1^ ^]^ With the rapid development of directed differentiation technology of cardiac cells from human pluripotent stem cells (hPSCs), cell therapy focusing on the transplantation of CMs has emerged as a promising strategy.^[^ ^2^ ^]^ However, the harsh microenvironment of the injured myocardium hindered the cellular engraftment of implanted cells,^[^ ^3^ ^]^ which is mainly caused by the poor nutritional and oxygenous supply in the tissue as ischemic areas are lack of vasculature.^[^ ^4^ ^]^ On the other hand, engineered cardiac tissues especially large and thick cardiac graft also need well‐organized vascular networks to provide oxygen and nutrients to all living cells.^[^ ^5^ ^]^ In fact, while the human heart has a dense vessel network of 2000–3000 capillaries mm^−2^, currently implantable cardiac constructs have ≈7‐fold lower microvascular density than that of the native heart.^[^ ^6^ ^]^ Thus, therapeutic angiogenesis especially in vitro fabrication of pre‐vascularized cardiac tissues with well‐organized and high‐density microvasculature is of vital importance to promoting retention and survival rate of transplanted cells.

Since blood vessels are developed from specific vascular progenitor cells which incorporate into the developing organ and self‐organize into the vascular system (including vascular arteries, veins and capillary network), engineering vascularized tissues yet remains very challenging.^[^ ^7^ ^]^ Vascular populations from hPSCs have been defined to undergo morphogenesis and self‐organize to form microvascular networks in an engineered matrix.^[^ ^8^, ^9^ ^]^ Recent studies have reported the development of self‐organizing 3D human blood vessel organoids which contain endothelial cells and pericytes that self‐assemble into capillary networks in extracellular matrix (ECM) hydrogel.^[^ ^10^ ^]^ ECM of capillaries such as collagen and fibronectin provide structural support to microvascular cells and serve as an essential component for proper capillary morphogenesis.^[^ ^11^ ^]^ Fabrication of cardiac tissues by means of molding in 3D hydrogel^[^ ^12^, ^13^ ^]^ or 3D bioprinting with arranged hydrogel^[^ ^14^, ^15^ ^]^ can construct myocardial tissue without rigid biomaterial, and can offer similar microenvironment to natural cardiac tissue. However, it has not been clarified whether the self‐assembly of vascular progenitor cells in ECM hydrogel can contribute to fabricating cardiac tissues with well‐organized microvasculature. Recent studies have developed cardiac tissues with limited microvasculature by co‐culturing with endothelial cells in small scale,^[^ ^16^, ^17^, ^18^ ^]^ or by three‐dimensional (3D) bioprinting of CMs and endothelial cells to construct centimeter‐scale cardiac patches.^[^ ^19^, ^20^ ^]^ But these studies taking advantage of terminally differentiated endothelial cells had not generated well‐organized and high‐density microvasculature, and the engraftment was depending on stimulating limited host vessel invasion rather than on direct vascular network formation by transplanted cells.

In this work, we identified human embryonic stem cells (hESCs) derived early vascular cells (EVCs) as seeding cells for vascular engineering in cardiac tissues. Firstly, EVCs were proved to be essential for successful differentiation of microvascular cells and self‐assembly of microvascular networks in ECM hydrogel. Then, cardiac microtissues with well‐organized and high‐density microvasculature were fabricated by 3D co‐culture of EVCs and CMs. Furthermore, taking advantage of in situ differentiation and self‐assembly of EVC spheroids, we developed centimeter‐scale cardiac patches with well‐organized microvasculature by 3D bioprinting technology. More importantly, we proved that EVC spheroids are superior to EVC single cells in engineering vascularized cardiac tissues and more helpful in cardiac function recovery after transplantation in mouse MI models. This study will provide transformative information that may contribute to develop the promising cell therapies for myocardial infarction.

## 2 Results

### 2.1 hESCs‐Derived EVCs Self‐Assemble into 3D Vascularization in ECM Hydrogel

**Figure 1:**
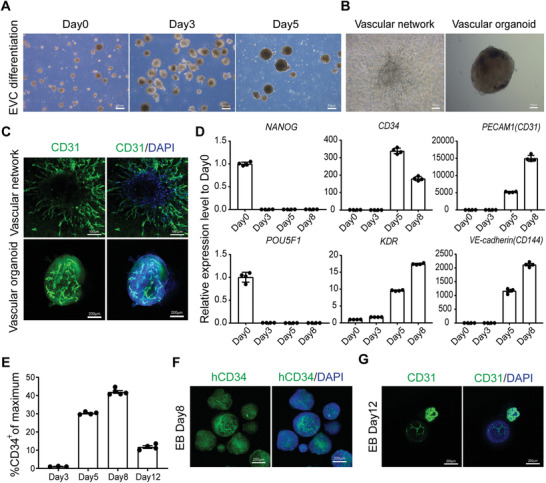
3D differentiation and verification of self‐assembly ability of early vascular cells (EVCs) from human embryonic stem cells (hESCs). A) Bright field images of embryoid body (EB) based differentiation of EVCs from hESCs line H9 at different stages. Scale bars, 200 µm. B) Bright field images of vascular network and vascular organoid constructed after embedding of EBs into Collagen I‐Matrigel matrix. Scale bars, 100 µm. C) Representative immunofluorescence (IF) staining of CD31^+^ endothelial cells indicated establishment of vascular networks and vascular organoids. Scale bars indicate 100 and 200 µm, respectively. D) qPCR analysis of stage‐specific genes at the indicated time point of EB‐based differentiation from H9, values represent expression level relative to Day 0 controls which were highly expressed with pluripotent genes *NANOG* and *POU5F1*, data were expressed as mean ± SEM (*n* = 4). E) The ratio of CD34^+^ cells in EVC EBs was identified by fluorescence activated cell sorting (FACS) analysis at the indicated time of EB‐based differentiation from H9, data were expressed as mean ± SEM (*n* = 4). F) IF staining showed high ratio of hCD34^+^ cells in Day 8 EBs. Scale bars, 200 µm. G) IF staining of CD31^+^ endothelial cells in EBs without embedding in Collagen I‐Matrigel matrix. Scale bars, 200 µm. EB, embryoid body; EVC, early vascular cell.

Previous studies have reported the development of self‐organizing 3D human blood vessel organoids from hPSCs, which contain endothelial cells and pericytes that self‐assemble into capillary networks.^[^ ^10^, ^21^ ^]^ Intrigued by this, we also developed a 3D differentiation method by stage‐specific modulating of mesoderm and vascular lineage development with hESCs‐derived embryoid bodies (EBs) (**Figure** **1**A). Quantification of EB size using NIS‐Elements software (Nikon) revealed that EBs grow from Day 0 to Day 5, EVCs at Day 5 have an average equivalent diameter of 144.63 µm (Figure S1A, Supporting Information). Embedding of EBs at Day 5 into Collagen I‐Matrigel matrix resulted in the self‐organizing of vascular networks and we further fabricated vascular organoids by dissection of these vascular networks (Figure 1B), which were shown to express endothelial cell specific CD144 by vital staining (Figure S1B, Supporting Information). Immunofluorescence (IF) staining and laser confocal microscopy further demonstrated that cells in vascular networks and vascular organoids expressed endothelial specific marker CD31 and interconnected to form complex networks (Figure 1C). 3D reconstitution of CD31^+^ endothelial cells in vascular organoids also showed that endothelial cells self‐assemble into well‐organized vascular networks (Movie S1, Supporting Information).

As EB‐based differentiation is stage‐specific and vascular endothelial cells are developed from multipotent progenitor cells, we assume that vascular progenitors such as EVCs contribute to the 3D self‐assembled vascularization in these multistep procedures. To analyze stage‐specific markers of vascular lineage development from hESC line H9, we performed real‐time PCR analysis during differentiation stages and demonstrated that Day 5 of differentiation represented the onset of EVCs specialization as markers such as CD34 and KDR started to express, and afterwards endothelial specific CD31 and CD144 were highly expressed, indicating the successful differentiation of EVCs (Figure 1D). Accordingly, fluorescence activated cell sorting (FACS) analysis revealed that EBs at Day 5 and Day 8 have high ratio of CD34^+^ endothelial progenitor cells, which were 30.28% and 41.98%, respectively (Figure 1E). This was also supported by IF staining of CD34 in EBs at Day 8 (Figure 1F). Meanwhile, EBs at Day 8 expressed endothelial markers of CD31 and CD144 (Figure 1D), which indicated the progressive induction of endothelial cells from EVCs during EB‐based differentiation. Importantly, we also proved that the generated vascular organoids consist not only CD31^+^ endothelial cells, but also PDGFR*β* ^+^ pericytes, which were in close interconnection, as shown in the orthogonal projection of immunofluorescence staining (Figure S1C, Supporting Information). All these results indicated that EBs at Day 5 consist EVCs which can further differentiate into vascular cells and form vasculature. We further studied whether EBs at Day 5 can develop into well‐organized microvasculature without matrix embedding. As shown in Figure 1G, confocal images exhibited that without matrix embedding, although CD31 was expressed in these differentiated EBs, CD31^+^ endothelial cell sprouting and network formation were not well‐organized. Thus, we have successfully fabricated 3D vascular networks and proved that in situ differentiation and self‐assembly of EVCs in ECM hydrogel contribute to 3D vascularization.

### 2.2 EVCs Co‐Cultured with CMs Develop into Vascularized Cardiac Microtissues in ECM Hydrogel

**Figure 2:**
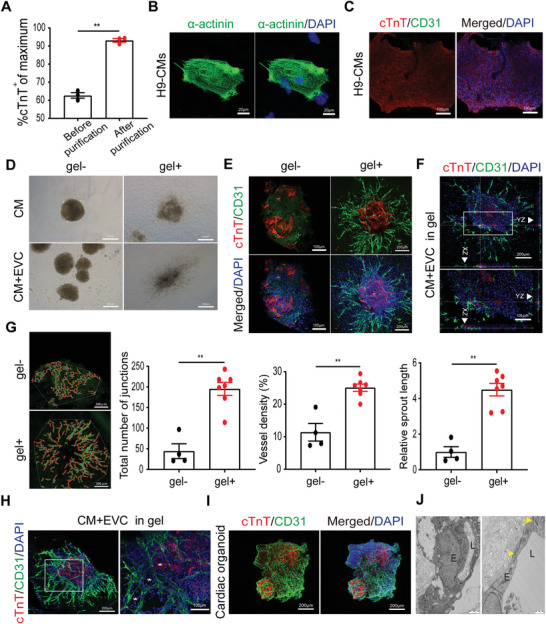
3D fabrication of vascularized cardiac microtissues by co‐culture of early vascular cells (EVCs) and cardiomyocytes (CMs) from hESCs. A) The ratio of cTnT^+^ CMs before and after lactate‐based purification was identified by fluorescence activated cell sorting (FACS) analysis on Day 12 of cardiac differentiation from H9 (*n* = 3). B) Representative immunofluorescence staining of *α*‐actinin showed striated sarcomere organization in H9‐derived CMs. Scale bars, 20 µm. C) Immunofluorescence (IF) staining of cTnT and CD31 showed high ratio of CMs and rare detection of endothelial cells after lactate‐based purification of cardiac differentiation from H9 hESCs. Scale bars, 100 µm. D) Bright field images of spheroids generated by aggregation of CMs with or without EVCs and their representative morphology after embedding in Collagen I‐Matrigel matrix. Scale bars, 200 µm. E) Detection of CD31^+^ endothelial cells sprouting by IF staining of cTnT and CD31 in cardiac spheroids embedded in Collagen I‐Matrigel matrix or not. Scale bars indicate 100 and 200 µm, respectively. F) Orthogonal projection of cTnT and CD31 staining in cardiac spheroids embedded in Collagen I‐Matrigel matrix. Scale bars indicate 200 and 100 µm, respectively. G) Quantitative analysis of cell sprouting in cardiac spheroids embedded in gel or not, representative analytic results using AngioTool software were shown on the left (*n* = 4). Scale bars, 200 µm. H) IF staining of cTnT and CD31 in spheroids embedded in gel, asterisks indicate bubble or loop like structure. Scale bars indicate 200 and 100 µm, respectively. I) IF staining of cTnT and CD31 in representative cardiac organoid. Scale bars, 200 µm. J) Representative transmission electron microcopy images of vascularized cardiac organoids. Note the generation of lumenized structure and the appearance of tight junctions (yellow arrowheads). Scale bars indicate 2 and 1 µm, respectively. Data in A and G were expressed as mean ± SEM and compared using independent sample *t*‐test. Statistical significance was defined as *p* < 0.05 (^**^ *p* < 0.01 or *p < 0.05). CM, cardiomyocyte; EVC, early vascular cell; gel‐, without embedding in gel; gel+, embedding in gel; E, endothelial cell; L, lumen.

As demonstrated above, EVCs could self‐assemble to form vasculature in ECM hydrogel. Therefore, we speculated that the incorporation of these progenitor cells with CMs may help to construct vascularized cardiac tissues with organized microvasculature in the same way as vascular organoids were constructed. Firstly, we generated H9‐derived CMs by performing monolayer‐based cardiac differentiation (Movie S2, Supporting Information) as previously reported^[^ ^22^, ^23^ ^]^ and confirmed the efficiency and quality of CMs by immunostaining of cardiac specific markers, e.g., cTnT and *α*‐actinin. To reduce interference from non‐CMs, we performed lactate‐based purification. As anticipated, FACS analysis of cTnT revealed that purity of CMs increases from 62.7% to 93.13% after purification (**Figure** **2**A). Immunofluorescence staining of cTnT and *α*‐actinin revealed that purified CMs had abundant myofilament expression (Figure S2A, Supporting Information). High magnification of *α*‐actinin staining further proved well‐organized sarcomere in CMs, indicating structural maturation of differentiated CMs (Figure 2B). Importantly, no CD31^+^ endothelial cells were detected in purified CMs (Figure 2C).

To verify whether a combined 3D culture of CMs with EVCs can develop into well‐organized microvasculature, we then co‐cultured CMs with EVCs by the hanging‐drop method to form cell aggregates. Firstly, we proved that dissociated EBs at Day 5 can re‐aggregate into spheroids and develop into vascular networks in ECM hydrogel (Figure S2B, Supporting Information). As anticipated, cell aggregates with CMs only showed no signs of vascular endothelial cell sprouting no matter with or without matrix embedding, while cell aggregates of CMs and EVCs showed evident sprouting after embedding in the matrix (Figure 2D), which were positively stained for endothelial specific CD31 (Figure S2C, Supporting Information). Meanwhile, we observed spontaneous beating of cell aggregates embedded in the matrix, indicating functional integrity of CMs after co‐culture with EVCs (Movie S3, Supporting Information).

To test whether spheroids of CMs and EVCs can form vascular structures without embedding in gel, we performed IF staining in these spheroids with or without embedding. As shown in Figure 2E, sprouting of CD31^+^ endothelial cells and signs of vascular networks were both detected in spheroids of CMs and EVCs with or without embedding, and spheroids embedded in gel showed strong evidence of CD31^+^ endothelial cells sprouting and organized vascular networks formation surrounding cTnT^+^ CMs. As the sprouting of CD31^+^ endothelial cells is radial in gel, we performed orthogonal projection of spheroids embedded in gel to see whether endothelial networks were forming over or through CM aggregate. Although we did not notice hollow like structures formed by endothelial cells, our results indicated that sprouted CD31^+^ endothelial cells and cTnT^+^ CMs are in close association, the inner endothelial cells are likely sprouting through the CM aggregate (Figure 2F). Further quantitative analysis measured from CD31^+^ staining images also backing our observation that the total number of junctions, vessel density, and relative sprout length were all significantly elevated after embedding of spheroids in gel (Figure 2G), indicating organized vasculature can form better after embedding in gel. Besides, CD31^+^ endothelial cells were found to form well‐organized vascular networks with bubble or loop like structures in these embedded spheroids, indicating self‐assembled vascularization with organized structure in cardiac microtissues (Figure 2H). When isolated from gel, the‐obtained 3D cardiac microtissues with vascular networks can further develop into vascularized cardiac organoids (Figure S2D, Supporting Information), which were beating spontaneously (Movie S4, Supporting Information) and exhibited high‐density and well‐organized CD31^+^ vascular networks spreading across the entire cardiac tissue (Figure 2I). Transmission electron microscope of these cardiac organoids revealed that continuous lumens enveloped by endothelial cells have generated, also with appearance of tight junctions (Figure 2J). Thus, we have proved that taking advantage of the self‐assembly ability of EVCs in ECM hydrogel can help to fabricate vascularized cardiac tissues by 3D co‐culture with CMs and in situ differentiation.

### 2.3 EVC Spheroids Promote Self‐Assembled Vascularization in 3D Bioprinted Cardiac Patch

**Figure 3:**
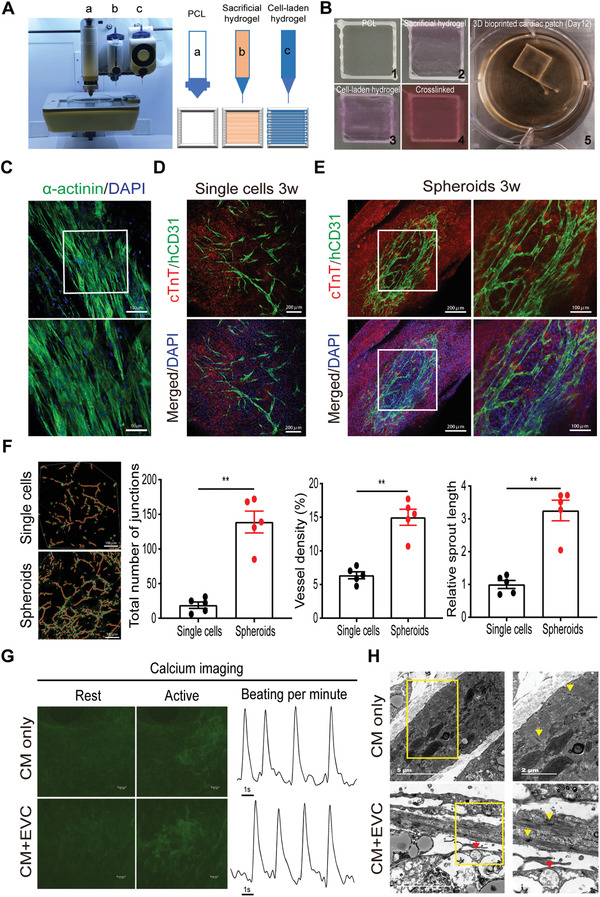
Spheroids of early vascular cells (EVCs) improve vascularization in 3D bioprinted centimeter‐scale cardiac tissues. A) Representative and schematic images of BIO‐X 3D bioprinter for bioprinting cardiac tissue. Typical three components of the bioprinter were marked as a, b, and c. Thereinto, “a” represents thermoplastic printhead, “b” represents conventional pneumatic printhead and “c” represents temperature‐controlled printhead. B) Optical images of procedures for the bioprinted cardiac tissue were shown. The size of poly (e‐caprolactone) (PCL) frame is 1 × 1 cm. 1–5 represent the sequential procedures of bioprinting process. C) Representative immunofluorescence (IF) staining of *α*‐actinin in bioprinted rat cardiomyocytes (CMs). Scale bars indicate 100 and 50 µm, respectively. D) IF staining of cTnT and human CD31 in cardiac patch with EVC single cells. Scale bars, 200 µm. E) IF staining of cTnT and human CD31 in cardiac patches with EVC spheroids and cultivated for 3 weeks showed organized vascular networks. Scale bars indicate 200 and 100 µm, respectively. F) Quantitative analysis of endothelial cell sprouting in cardiac patches incorporated with EVC single cells or spheroids, representative analytic results using AngioTool software were shown on the left (*n* = 5). Scale bars, 100 µm. Data were expressed as mean ± SEM and compared using independent sample *t*‐test. Statistical significance was defined as *p* < 0.05 (^**^ *p* < 0.01 or *p < 0.05). G) Representative calcium images at rest and active stages and the synchronized contraction was shown by calcium flux frequency. H) Representative transmission electron microcopy images of 3D bioprinted cardiac patches incorporated with or without EVC spheroids. Note the generation of lumenized structure (red arrowheads) and the appearance of intercalated discs (yellow arrowheads). Scale bars indicate 5 and 2 µm, respectively. CM, cardiomyocyte; EVC, early vascular cell.

To date, the focus on replicating complex and heterogeneous tissue constructs continues to increase as 3D bioprinting technologies advance.^[^ ^24^ ^]^ Prevascularized patches can enhance cell survival and engraftment rate after transplantation. To explore whether the self‐assembling strategy could facilitate the fabrication of microvasculature with cellular organization in matured and functional cardiac tissues, we applied 3D bioprinting system to construct centimeter‐scale 3D cardiac patches. In this study, we used previously reported multimaterial 3D bioprinting strategy to construct cardiac tissue that is anchored to a plastic poly (e‐caprolactone) (PCL) frame, as well as insulated from the culture surface by a layer of sacrificial hydrogel.^[^ ^25^ ^]^ Briefly, the cardiac patch was printed via BIO‐X 3D Bioprinter as designed and sequentially processed layer‐by‐layer with thermal PCL polymer, sacrificial hydrogel, and cell‐laden hydrogel (**Figure** **3**A). In this study, we combined primary CMs isolated from infant rat hearts with EVCs differentiated from hESCs and suspended together in a fibrin‐based bioink. The cell‐laden hydrogel was then printed as designed to fabricate into patch form and cultured under vascular lineage inducing factors to promote in situ vasculature differentiation. Optical images during procedures in cardiac tissue bioprinting were shown to indicate structures before and after the 3D bioprinting process (Figure 3B). Firstly, live/dead cell staining was used to assess the viability of bioprinted CMs in cardiac patches after the 3D bioprinting process, although some dead cells were shown on post‐printing Day 1, bioprinted CMs cultured for 1 week aligned tightly with rare dead cells detected (Figure S3A, Supporting Information). Besides, as shown in Figure 3C of *α*‐actinin IF staining, bioprinted CMs possessed aligned myofilaments and bead‐like sarcomere structure after 3 weeks cultivation, indicating the CMs were in mature state.

Furthermore, to analyze whether EVCs can differentiate and self‐assemble into microvasculature in centimeter‐scale cardiac tissue, we combined rat CMs with EVCs in single cell form or spheroids to print 3D cardiac patches. Considering the bioprinting process may be detrimental, we firstly applied live/dead cell staining assay to monitor the viability of EVC spheroids before and after the bioprinting process. AO‐PI staining showed that EVC spheroids remain viable and even more compact in the printed cardiac patch, indicating the stress during bioprinting and subsequent cross‐link of hydrogel had minor side effect on cell survival (Figure S3B, Supporting Information). Further study using IF staining of human specific CD31 showed that in groups using EVC single cells as seeding cells, endothelial cells were evenly distributed and sprouted in the constructed cardiac tissue after 3 weeks cultivation, but there were no signs of evident vascular networks formation (Figure 3D). But in groups using the same amount of EVC spheroids as seeding cells, IF staining suggested that hCD31^+^ endothelial cells were not dispersive in CMs, and spheroids in the cardiac patch can develop into spider‐like vascular structures (Figure S3C, Supporting Information). Besides, these bioprinted patches showed evident and high‐density vascular network formation which were interconnected and spreading across the cardiac tissue even in the early weeks of cultivation, as shown by hCD31 and cTnT staining (Figure S3D, Supporting Information). More importantly, these vascular networks have developed more conjunct and interconnected as cultivation prolonged from 1 week to 3 weeks, which indicated the in situ differentiation and self‐assembling of EVC spheroids (Figure S3D, Supporting Information and Figure 3E). Meanwhile, well‐organized vascular networks generated from EVC spheroids can also be detected in these cardiac patches (Figure 3E and Movie S5, Supporting Information). Orthogonal projection of IF staining in cardiac patch with CMs and EVC spheroids further proved that vascular lumens enveloped by hCD31^+^ endothelial cells have generated (Figure S3E, Supporting Information). Quantitative analysis measured from CD31^+^ staining images revealed that the total number of junctions, vessel density, and relative sprout length were all significantly higher in groups of cardiac patches incorporated with EVC spheroids (Figure 3F), indicating organized vasculature can form better using spheroids rather than single cells. Importantly, incorporation of EVC spheroids with neonatal rat CMs did not change the spontaneous beating property of the 3D bioprinted cardiac patch (Movies S6 and S7, Supporting Information). In addition, calcium imaging revealed that neonatal rat CMs in the printed cardiac patch have synchronous contraction as calcium influx spreads across the entire patch, and the incorporation of EVC spheroids in the cardiac tissue did not change the calcium influx frequency (Figure 3G), representative calcium imaging of the 3D bioprinted cardiac tissues with or without EVC spheroids were shown in Movies S8 and S9 (Supporting Information), all these results indicated that the 3D bioprinting strategy does not influence the function of matured CMs after 3 weeks cultivation. Transmission electron microscope of 3D bioprinted cardiac patches with or without EVC spheroids revealed that lumens enveloped by endothelial cells can be generated, and aligned sarcomere structure with intercalated discs can be detected (Figure 3H). Together, we have fabricated functional cardiac patches with well‐organized and high‐density microvasculature by 3D bioprinting with CMs and EVC spheroids mediated vascular differentiation and self‐assembling.

### 2.4 EVC Spheroids Improve Cardiac Function in Mouse MI Model

**Figure 4:**
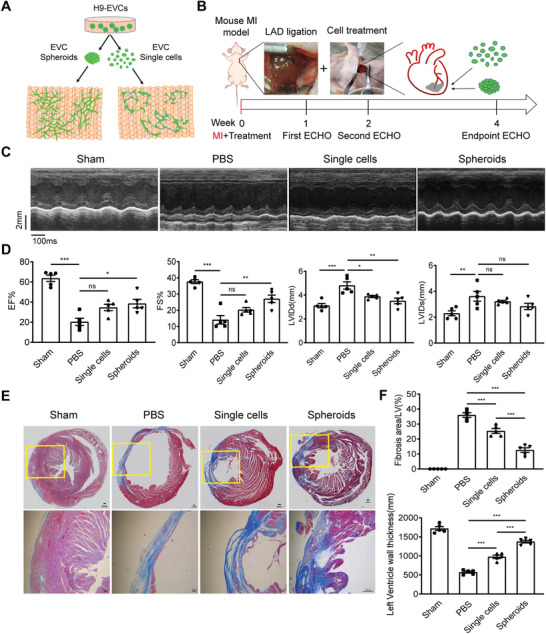
Transplantation of early vascular cell (EVC) spheroids improves cardiac function and reduces cardiac fibrosis after myocardial infarction (MI). A) Schematic overview of vascularized cardiac tissue engineering with EVC spheroids or EVC single cells, indicating vascularization of EVC spheroids is superior to EVC single cells. B) Schematic protocol for creation of mouse MI model and echocardiography (ECHO) detection. MI was induced by permanent ligation of the left anterior descending (LAD) coronary artery of nude mouse with a 8‐0 silk suture. Mouse underwent LAD ligation was immediately treated via intramyocardial injection with EVC spheroids or equal amount of 1 × 10^6^ singularized EVCs (EVC single cells). Cardiac function was assessed longitudinally via ECHO analysis according to three time points: 1‐week, 2‐week, and 4‐week post‐MI. Histology was performed at 4‐week after treatment. C) Representative images of ECHO after MI for 4 weeks in different groups (*n* = 5 mice per group). D) Echocardiographic measurements of cardiac functions at 4 weeks post‐MI and cell transplantation (*n* = 5 mice per group). E) Representative images of Masson staining after MI for 4 weeks in different groups (*n* = 5 mice per group). F) Assessment of fibrosis area and comparison of left ventricle wall thickness (*n* = 5 mice per group). Data in D and F were shown as mean ± SEM and analyzed using one‐way ANOVA followed by Tukey's post‐hoc analysis. Statistical significance was defined as *p* < 0.05 (^***^ *p* < 0.001, ^**^ *p* < 0.01 or *p < 0.05; ns means no significance). MI, myocardial infarction; LAD, left anterior descending; ECHO, echocardiography; EF, ejection fraction; FS, fractional shortening; LVIDd, left ventricular internal dimension‐diastolic; LVIDs, left ventricular internal dimension‐systolic.

The in vitro studies revealed that EVC spheroids can be superior to EVC single cells in engineering vascularized cardiac tissues, especially in the fabrication of centimeter‐scale cardiac patch (**Figure** **4**A). On the other hand, the very low retention rate of single‐cell suspensions after delivery is an obstacle for cell therapy, transplantation using spheroids or microtissues has emerged as an encouraging alternative.^[^ ^26^ ^]^ We assumed that EVC spheroids may also be superior to EVC single cells in cell engraftment and vascularization in vivo, especially in the treatment of myocardial infarction. To test this hypothesis, we created nude mouse MI models by ligation of the left anterior descending (LAD) coronary artery and injected hESCs‐derived EVCs as spheroids or single cells at the border zone via intramyocardial strategy (Figure 4B).

To evaluate the cardiac functional recovery, transthoracic echocardiography (ECHO) was performed at 1, 2, and 4 weeks after the treatment (Figure 4B). ECHO measurements of cardiac functions showed successful ligation in MI and cell transplantation groups at 1 week post‐MI (Figure S4A, Supporting Information). Statistical analysis at 1 week also revealed MI injury occurred after LAD ligation in groups of MI control group, single cells treated group, and spheroids treated group (Figure S4B, Supporting Information). The representative echocardiographic data at 4 weeks post‐MI suggested that cardiac function of MI treated groups with EVC single cells or EVC spheroids were all improved (Figure 4C). Statistical analysis of echocardiographic data at 4 weeks revealed a significant improvement of ejection fraction (EF) in EVC spheroids treated groups (≈38.51%) compared to MI control groups (≈20.35%), meanwhile, EF in EVC single cells treated groups increased to ≈34.66%. And delivery of EVC spheroids resulted in reversal of the fractional shortening (FS) loss (≈27.05%) when compared to MI control groups (≈14.14%), which also indicated an improvement in cardiac function. Besides, left ventricular internal dimension‐diastolic (LVIDd) was significantly decreased in EVC spheroids and EVC single cells treated groups compared to MI control groups. While the decrease of left ventricular internal dimension‐systolic (LVIDs) in treated groups was not significant compared to MI control (Figure 4D).

Then, we performed histochemical stain to investigate whether injection of EVCs attenuates cardiac fibrosis. Masson staining of frozen sections was used to detect cardiac fibrosis area in different groups, which suggested that fibrosis area in treated groups is smaller than MI control (Figure 4E). Statistical analysis also revealed that the rate of fibrosis area in spheroids treated groups significantly decreased compared to single cells treated groups and MI control, accordingly, left ventricle wall thickness was also significantly improved in EVC spheroids treated groups (Figure 4F). Besides, we have tried to detect changes in tissue vascularization in vivo from frozen sections of the excised hearts, but regrettably, we could not detect and image the appropriate sections where the treatment is injected. Our results indicate that to a certain extent, transplantation of EVC spheroids may help to cardiac function recovery after MI.

## 3 Discussion

Blood vessels play vital roles in organ development and disease progress such as ischemic heart disease, stroke, and cancer.^[^ ^21^ ^]^ Since the development of hPSCs technology, engineering of vascularized tissues has opened up great potentials for regenerative medicine.^[^ ^27^ ^]^ However, current knowledge about how to re‐vascularize tissues is limited owing to a lack of understanding of how organs are normally vascularized during development.^[^ ^4^ ^]^ Recently, Colunga et al. proved that multipotent vascular progenitor cells generated from hPSCs contribute to vascular lineage differentiation and neovascularization of damaged tissue, efficiently populate vascular scaffolds and self‐assemble into functional vessels.^[^ ^28^ ^]^ Wimmer et al. reported the development of self‐organizing 3D human blood vessel organoids containing capillary networks from hPSCs.^[^ ^10^ ^]^ In this work, we re‐established these vascular organoids in our lab and identified that EVCs containing CD34^+^ progenitor cells exist in the vascular induction stage, which was essential for the successful differentiation of microvascular cells (CD31^+^ endothelial cells and PDGFR*β* ^+^ pericytes) and the self‐assembly of microvascular networks in ECM hydrogel.

Angiogenesis involves interplay between the native ECM, vascular endothelial cells, and growth factors.^[^ ^29^ ^]^ hPSCs‐derived EVCs are well‐defined to mature into endothelial cells and pericytes, which can self‐organize to form microvascular networks in engineered matrix.^[^ ^8^ ^]^ And the proven capability of EVCs to assemble into 3D networks in engineered matrices also indicated that hydrogel is essential for successful self‐organized vascular network formation.^[^ ^9^ ^]^ This is of great importance for vascularized tissue engineering which reminds us to fabricate vascularized cardiac tissues by co‐culture of EVCs with CMs in ECM hydrogel to promote self‐assembled vascularization. In this work, we proved that co‐culture strategy in ECM hydrogel indeed promoted the self‐assembly of microvascularization in cardiac microtissues and we further developed vascularized cardiac organoids with well‐organized microvasculature by referring to the strategy of vessel organoid construction.^[^ ^21^ ^]^ Apart from the well‐known Matrigel or Collagen matrix for tissue engineering, other ECM component also participates in angiogenesis.^[^ ^11^ ^]^ Our results further revealed that EVC spheroids can self‐assemble into microvasculature in fibrin hydrogel‐based 3D bioprinted cardiac patches, which also indicate that ECM hydrogel serves as an important factor in successful vascularization. In this work, the multipotent differentiation property of EVCs and 3D microenvironment created by hydrogel enables in situ differentiation and 3D self‐assembling of microvascular structures in cardiac organoids and cardiac tissues. Although former studies had used co‐culture strategy to fabricate micro heart tissues,^[^ ^16^, ^17^, ^30^ ^]^ most of them used endothelial cells rather than vascular progenitor cells, and few of them had applied hydrogel to create 3D environment for self‐assembly. Without in situ differentiation and self‐assembly, the generated microvasculature in these cardiac tissues was not well‐organized.

Preclinical studies have shown that transplantation of hPSCs‐derived CMs has limited effect on myocardium regeneration and cardiac functional recovery which was due to the low survival and engraftment rate of injected cells,^[^ ^31^ ^]^ alternatively, fabrication of cardiac graft offers a promising strategy for cell therapy.^[^ ^13^ ^]^ However, lack of vascularization in engineered cardiac patches hindered the clinical application to treat ischemic heart disease, as the survival and retention of CMs remain an essential problem, especially in large and thick cardiac patches. 3D bioprinting can arrange cells and ECM at the micron scale, structure, and function of 3D bioprinted cardiac patches with hPSCs‐derived cardiovascular cells more closely resemble natural myocardial tissues compared to those created by traditional tissue engineering.^[^ ^14^ ^]^ In this study, we applied anchorage dependent cardiac tissue printing strategy to construct cardiac patches and incorporated EVC spheroids within the cell‐laden hydrogel, which were induced differentiation in situ and self‐assembled into microvasculature in the contractile cardiac tissue with cellular organization. Although 3D bioprinting technology has already been used to develop large grafts by co‐culturing of endothelial cells and CMs, these endothelial cells are lack of engraftment with CMs,^[^ ^19^ ^]^ our strategy using the self‐assembly of EVC spheroids allowed us to establish well‐organized and high‐density microvasculature in centimeter‐scale cardiac tissues, which has great potential for cell‐based therapy of MI.

Although traditional tissue engineering methods such as surface seeding or hydrogel molding have been widely used to construct myocardium, they often result in less organized cardiac tissues. In these models, the surfaces are either too rigid for cardiomyocytes to transmit contraction stress effectively,^[^ ^32^ ^]^ or too compartmentalized to develop into a single piece of macroscopic synchronous contractile myocardium with cardiomyocytes aligned resembling native cardiac tissue.^[^ ^30^, ^33^, ^34^ ^]^ In this study, we used multimaterial 3D bioprinting to construct cardiac tissue that is anchored to a PCL frame, as well as insulated from the culture surface by a layer of sacrificial hydrogel. Consequently, the engineered cardiac tissue builds up internal tension while contracting against the relatively rigid frame, allowing the cardiomyocytes to align and develop synchronous beating under mechanical stimulation. On the other hand, recent studies have reported cardiac patch models that could also develop into mature cardiac tissue with macroscopic and synchronous contraction using molding of cell‐laden hydrogels.^[^ ^35^, ^36^ ^]^ However, vascularization, especially vascularization via in situ differentiation, has yet been achieved in these models to our knowledge. More importantly, molding by injection requires hydrogels to be at low‐viscosity to form a smooth layer in the mold. When mixed and extruded with such thin fluid, the EVC spheroids would sink to the bottom of the construct rapidly due to gravitational force and result in inhomogeneous construct. With our temperature‐controlled 3D bioprinting strategy, cell/spheroid‐laden hydrogel can be extruded at a relatively high viscosity so that the construct will remain relatively homogeneous in terms of spatial distribution of EVC spheroids. Limited to the positional accuracy of spheroid bioprinting, one of the limitations in this approach is that EVC spheroids were randomly distributed inside the gel, which will result in nonuniformities in the tissue density and vascularization. This may be improved by referring to a recent approach which enabled picking and precise positioning of spheroids in 3D space by aspiration‐assisted bioprinting, and which can be used to bioprint EVC spheroids into the fibrin constructs before cross‐linking.^[^ ^37^ ^]^ On the other side, although most of our EVC spheroids have an equivalent diameter of 144.63 µm, spheroids with homogeneous and optimal size maybe more superior for 3D bioprinting, aggregate formation using U‐shaped ultralow‐attachment 96‐well plate can be applied in the future.

More importantly, in vitro studies in this work indicated that EVC spheroids are superior to EVC single cells in engineering vascularized cardiac tissues. Apart from engineering pre‐vascularized cardiac engraftment, cell therapy strategy with cell sheets or cell aggregates rather than singularized cells can also boost cell engraft rate and survival rate in injured heart.^[^ ^13^, ^38^ ^]^ Preclinical study has shown that MI therapy with hESCs‐derived CMs in non‐human primates has a very low survival rate (7%), which is in part due to the high pressure and continuous contraction of the pumping heart.^[^ ^31^ ^]^ Alternatively, cell delivery with cell sheets or cell aggregates can be more effective.^[^ ^13^, ^38^ ^]^ We assumed that EVC spheroids may also be superior to EVC single cells in the treatment of myocardial infarction. Our results indicated that to a certain extent, EVC spheroids may be more helpful for cardiac function recovery after injection into infarcted area, which suggested that cell aggregates can be an alternative cell delivery strategy for cell therapy. At the same time, we have also tried to evaluate the distribution and vascular differentiation properties of transplanted EVCs in infarcted area, we performed abundant IF staining analysis of frozen sections but unfortunately, we could not anchor to the precise cell engraftment position and we did not gain credible evidence of vascularization within the implanted area (data not shown). This is probably because of the complexity of the in vivo structure and microenvironment, which is also a common problem to be solved for cell tracing in cell therapy studies.^[^ ^3^ ^]^

The absence of a vascular system is deemed detrimental to organoids which are millimeter‐scale and undergo long‐term culture, our strategy may offer a method to vascularize organoids and to prevent apoptotic cell death at the inner‐most region because of limited oxygen and nutrient diffusion. On the other hand, understanding human heart development and cardiac disease mechanisms has been challenging due to the paucity of available tissues. In vitro fabrication of patient‐specific vascularized cardiac tissues with high‐fidelity using our strategy could have a transformative impact on drug screening and cardiac disease modeling.

## 4 Conclusion

In summary, we have successfully engineered cardiac tissues with well‐organized and high‐density microvasculature taking advantage of hESCs‐derived EVCs. And we have shown for the first time that EVC spheroids are superior to form 3D vascular networks than EVC single cells in 3D bioprinted myocardial tissues. Furthermore, transplantation of EVC spheroids may be more helpful as treated infarcted mice exhibit reduced infarct area and ameliorative cardiac function compared with control animals. We believe that this study will contribute to our understanding how EVC spheroids rebuild microvasculature in cardiac tissues. Therefore, this study offered critical and valuable information such as EVCs spheroids derived from hESCs can be used as one of seeding cells to rebuild the microvascular network, help improve the survival of transplanted cells, and may further improve cell therapy of myocardial infarction.

## 5 Experimental Section

### Human ESC Culture and Vascular Network Differentiation

Human ESC line H9 (WA09) was obtained from WiCell. For feeder independent and monolayer‐based culture, H9 was maintained on human qualified Matrigel (BD Biosciences) in mTeSR1 medium (STEMCELL Technologies) and was tested for mycoplasma contamination on a regular basis. EB‐based vascular differentiation was modified from method as previously described.^[^ ^21^ ^]^ Briefly, H9 colonies were dissociated with Accutase (Life Technologies) for 3 min, then 4 × 10^5^ cells were resuspended in mTeSR1 medium with 10 × 10^−6^ m Y‐27632 (Selleck Chemicals) and plated into one well of ultra‐low attachment 6‐well plate (Corning) for cell aggregation. Cell aggregates were then treated at Day 0 with 12 × 10^−6^ m CHIR99021 (Selleck Chemicals) and 30 ng mL^−1^ BMP4 (R&D systems) for mesoderm induction in RPMI1640 medium with B27 supplement minus insulin (RPMI/B27‐I) (Invitrogen), and at Day 3 with 100 ng mL^−1^ VEGF‐A (Peprotech) for vascular differentiation in EGM2 medium (Lonza). Then cell aggregates at Day 5 were embedded into Collagen I‐Matrigel matrix (Invitrogen) and overlaid with EGM2 medium containing 100 ng mL^−1^ VEGF‐A, 100 ng mL^−1^ FGF‐2 (Peprotech), and 10 × 10^−6^ m SB43152 (Selleck Chemicals) to increase the yield of endothelial cells and sprouting of vascular networks. At Day 10vascular networks were established and either directly analyzed or extracted from the matrix and further cultured in 24‐well low‐attachment plates (Corning) to self‐assemble into vascular organoids.

### Vascular Organoids Extraction and Cultivation

The extraction method refers to the generation of blood vessel organoids from hPSCs.^[^ ^21^ ^]^ Briefly, under a sterile condition, entire matrix containing vascular networks was liberated from the well bottom by round‐shaped spatula. Then the gel was transferred to the lid of a 10‐cm dish and vascular networks were cut out by two sterile 30‐gauge needles to remove excess matrix under a stereomicroscope (Nikon). Single vascular organoids were transferred by a cut P1000 tip and further cultured with EGM2 medium containing 100 ng mL^−1^ VEGF‐A, 100 ng mL^−1^ FGF‐2 (Peprotech), and 10 × 10^−6^ m SB43152 (Selleck Chemicals) in 24‐well low‐attachment plates (Corning) for another 4 days to promote self‐assembling. When round and fully encapsulated morphology of vascular organoids was established, floating vascular organoids can then be cultured up to 1 month.

### Monolayer‐Based Cardiac Differentiation

For monolayer‐based cardiac differentiation, singularized hESCs (H9) dissociated with Accutase were maintained on Matrigel in mTeSR1 medium for at least one generation, and differentiated into CMs via small molecule modulating of Wnt signaling. Briefly, when cells reached 80–90% confluence, culture medium was changed to RPMI/B27‐I and 6 × 10^−6^ m CHIR99021 for 2 days. The medium was replaced at Day 2 with RPMI/B27‐I for 1 day. And changed to RPMI/B27‐I with 5 × 10^−6^ m IWP2 (Selleck Chemicals) for 2 days. Cells were continued to cultivate in RPMI/B27‐I for 2 days, finally maintained in RPMI1640 medium with B27 supplement (RPMI/B27+I) (Invitrogen) until applied in related experiments (changing medium every 3 days). For lactate‐based CMs purification, differentiated cells were cultured in glucose‐depleted culture medium with 1 mmol L^−1^ lactate as the only carbon source for 7 days as previously reported.^[^ ^39^ ^]^

### Vascularized Cardiac Organoid Fabrication

On the day of cardiac spheroid formation, H9‐derived CMs and human EVC spheroids at Day 5 of differentiation were dissociated with Accutase and resuspended in RPMI/B27+I medium or EGM2 medium, respectively. Then cardiac spheroids consisting of CMs and EVCs were generated by hanging drop technique. In brief, each spheroid was generated from 10 000 cells at a ratio of CMs:EVCs (7:3) in 20 µL RPMI/B27+I:EGM‐2 (1:1) medium, and cultured in a hanging drop for 2 days at 37 °C and 5% CO_2_. Then cardiac spheroids were embedded into Collagen I‐Matrigel matrix (12‐well plate) and overlaid with RPMI/B27+I:EGM‐2 (1:1) medium containing 100 ng mL^−1^ VEGF‐A, 100 ng mL^−1^ FGF‐2, and 10 × 10^−6^ m SB43152 to induce differentiation of endothelial cells and sprouting of vascular networks. Spontaneous beating cardiac microtissues with vascular networks were established and either directly analyzed or extracted from the matrix and further cultured in 24‐well low‐attachment plates to self‐assemble into vascularized cardiac organoids referring to the method of vascular organoids extraction and cultivation.

### IF Staining and Microscopy

Vascular networks in the matrix or vascularized cardiac tissues were fixed with 4% (vol/vol) paraformaldehyde (YEASEN) for 1 h at room temperature, then permeated and blocked with immunostaining blocking/primary antibody dilution solution (BBI Life Sciences) for 2 h at room temperature. The samples were then incubated with primary antibodies overnight at 4 °C as following: anti‐CD31 (Abcam, ab24590, 1:500), anti‐cTnT (Abcam, ab45932, 1:400), anti‐*α*‐actinin (Abcam, ab137346, 1:400), and anti‐human CD31 (Abcam, ab9498, 1:1000). Next day, samples were incubated with Alexa‐flour‐conjugated secondary antibodies (Life Technologies) for 2 h at room temperature. Nuclei were stained with 1 µg mL^−1^ DAPI (YEASEN) in PBS for 5 min at room temperature. Images were captured under inverted fluorescence microscope (Leica) or captured on a Leica SP8 confocal microscope with a 10× or 20× objective. For quantification of vascular sprouting and network formation, vascular structures were determined by CD31 staining, images were captured using Leica SP8 confocal microscope. The number of branch points, the average sprout length, and vessel density were quantified using the AngioTool software.

### Quantitative Real‐Time PCR

Total RNA was prepared with RNAprep pure cell RNA extraction kit (TIANGEN) and RNA (500 ng) was reverse transcribed into cDNA with FastKing RT Kit (with gDNase) (TIANGEN) as per manufacturer's instructions. Quantitative real‐time PCR was performed using SuperReal PreMix Plus (SYBR Green) (TIANGEN) on ABI ViiA7 Real‐Time PCR System. The relative RNA abundance was calculated with the 2^–ΔΔCt^ means and normalized with respect to GAPDH expression level. The primer sets are listed in Table S1 (Supporting Information).

### Fluorescence Activated Cell Sorting

H9‐derived EBs or CMs were dissociated into single cells by incubation with 0.25% Trypsin‐EDTA (Invitrogen) at 37 °C and fixed with 1% (vol/vol) paraformaldehyde for 15 min at room temperature. For human CD34 staining, cells were incubated with CD34‐FITC antibody (eBioscience) diluted in 100 µL per sample FACS buffer (PBS without Ca^2+^/Mg^2+^, 1% BSA) for 40 min at room temperature and washed twice with FACS buffer before analysis. CMs were incubated with cTnT antibody (Abcam, ab45932) diluted in FACS buffer containing 0.1% Triton X‐100 for 40 min at room temperature, washed twice with FACS buffer, centrifuged, and incubated with secondary antibody in dark for 20 min at room temperature. Cells were washed twice with FACS buffer, centrifuged, supernatant discarded, and resuspended in 400 µL FACS buffer for analysis. Data were collected on flow cytometer (Beckman Cytoflex) and analyzed by FlowJo V10.

### Isolation and Culture of Neonatal Rat CMs

All animal experimental protocols were approved by the Institutional Animal Care and Use Committee of Tongji University. CMs were isolated from 2‐day old neonatal rat hearts. Briefly, neonatal rat hearts were harvested, minced, and enzymatically dissociated by serial digestion with 0.25% trypsin and 0.125% collagenase IV (Sigma‐Aldrich). Digested cardiac cells were then harvested by filtration and centrifugation, resuspended, and cultured for 1 h in DMEM/F12 (Invitrogen) with 10% fetal bovine serum (FBS) to allow for attachment of non‐CMs. Enriched CMs in the cell suspension were then collected and cultured in Claycomb culture medium (Sigma‐Aldrich) supplemented with 10% FBS, 1% l‐glutamine, 10 mg mL^−1^ aprotinin, and 1% penicillin/streptomycin (Invitrogen).

### Preparation of Hydrogels for 3D Bioprinting

The fibrin‐based hydrogel was prepared by dissolving 20 mg mL^−1^ fibrinogen, 30 mg mL^−1^ gelatin, 20 mg mL^−1^ aprotinin, and 3 mg mL^−1^ hyaluronic acid (HA) in DMEM culture medium containing 1% penicillin/streptomycin. Sacrificial hydrogel was prepared by dissolving 10% gelatin in DMEM culture medium containing 1% penicillin/streptomycin. All materials were gently dissolved at 37 °C in a temperature‐controlled shaker and sterilized by filtration through a 0.2 µm filter. The cell‐laden hydrogel was prepared by mixing the fibrin‐based hydrogel with neonatal rat CMs at a density of 10^7^ cells mL^−1^. For the fabrication of vascularized cardiac patches, oversized EVC spheroids were firstly deserted by gravity sedimentation, and the remaining spheroids were divided evenly into two parts for preparation of EVC single cells and EVC spheroids. Then, 500 spheroids mL^−1^ of EVC spheroids or equivalent amount of EVC single cells were added to the CMs‐laden hydrogel. Cell viability in hydrogel was analyzed by AO‐PI staining kit (Nexcelom Bioscience) and captured using Leica SP8 confocal microscope. All materials mentioned were obtained from Sigma‐Aldrich unless stated otherwise.

### Fabrication and Culture of Cardiac Patches

The vascularized cardiac patches were fabricated according to a modified protocol based on a previous report.^[^ ^25^ ^]^ A BIO‐X 3D Bioprinter (CELLINK) was used to perform pneumatic‐extrusion with a thermoplastic printhead, a conventional pneumatic printhead, and a temperature‐controlled printhead. Customized G‐code was created to guide the motion and material dispensing of the 3D Bioprinter for the printing process. On the day of fabrication, PCL was loaded into the thermoplastic printhead which was set to 90 °C, 120 kPa pneumatic pressure, 60 mm min^−1^ printhead speed, and equipped with a 400 µm metal nozzle. The sacrificial hydrogel was loaded into the conventional pneumatic printhead which was set to 20 kPa pneumatic pressure, 100 mm min^−1^ printhead speed, and equipped with a 25G blunt needle. The cell‐laden hydrogel was loaded into the temperature‐controlled printhead which was set to 18 °C, 80 kPa pneumatic pressure, 100 mm min^−1^ printhead speed, and equipped with a 22G blunt needle. The printing platform was set to 10 °C. All parts and materials were equilibrated to the desired temperature for 30 min before the printing started.

To fabricate the cardiac patch, PCL was used to firstly print a 1× 1 cm square frame structure, which has poles on two opposite sides allowing the cell‐laden hydrogel to be anchored. Within the square frame, a layer of sacrificial hydrogel was disposed to insulate the cell‐laden hydrogel from the surface beneath. The cell‐laden hydrogel with or without EVC single cells or EVC spheroids was then dispensed in a layer of parallel strings across the poles on the PCL frame to form a patch. After fabrication, the patches were immediately immerged in cold PBS containing 50 U mL^−1^ thrombin and allowed to cross‐link for 20 min under room temperature. Cardiac patches containing CMs only were cultured in cardiac growth medium containing Claycomb medium supplemented with 10% FBS, 1% l‐glutamine, 10 mg mL^−1^ aprotinin, and 1% penicillin/streptomycin. Cardiac patches containing EVCs were firstly cultured for 7 days in 1:1 mixture of Claycomb medium (Sigma‐Aldrich) and EGM2‐medium (Lonza) supplemented with 100 ng mL^−1^ VEGF, 50 ng mL^−1^ bFGF, 10 × 10^−6^ m SB431542, 10% FBS, 1% l‐glutamine, 10 mg mL^−1^ aprotinin, and 1% penicillin/streptomycin. After 7 days, culture medium for cardiac patches containing human EVCs was replaced with cardiac growth medium. To stimulate the contraction of CMs, 200 × 10^−9^ m epinephrine was added to all samples after 3 days of culture. Cell culture medium was replaced every 3 days.

### Ex Vivo Imaging of Calcium Transients

Ex‐vivo imaging of calcium transients was performed to validate the synchronous contraction in the engineered cardiac tissue. Cardiac patches containing CMs only or CMs with EVC spheroids were imaged after 3 weeks in culture. The patches were washed with PBS and incubated with 3 × 10^−6^ m Fluo‐8 AM (Abcam) for 20 min at 37 °C. Then, the patches were washed with DMEM medium containing 10% FBS for three times and mounted onto a Leica inverted fluorescence microscope. Fluorescent videos of calcium transients were recorded by Faststone screen record software at 30 fps. Fluorescence intensity in the videos was analyzed with ImageJ software and plotted with GraphpadPrism software.

### MI Model and Animal Studies

All animal studies were approved by the Institutional Animal Care and Use Committee of Tongji University. In this study, nude mice were employed to minimize host immune rejection in animals after transplantation of human EVCs. Adult male nude mice (18–22 g) were purchased from Shanghai JieSiJie Laboratory Animals. MI was induced by permanent ligation of the LAD coronary artery with a 8‐0 silk suture. Immediately after MI induction, human EVC spheroids at Day 5 or equal amount of singularized EVCs (1 × 10^6^ cells) suspended in 10 µL PBS were injected into the border zone of infarction with U40 Insulin syringe (BD). Age‐matched nude mice with left thoracotomy only were used as sham group and mice after LAD ligation without cell injection were used as MI group. In each group, the cardiac function of the surviving mice was evaluated by means of ECHO according to three time points: 1‐week, 2‐week, and 4‐week post‐MI. Animals were sacrificed 28 days after human EVCs transplantation.

### Histological Analysis

For histochemical and immunohistochemical staining of cardiac tissues, 4 weeks after cell injection, the heart tissues from the nude mice were fixed with 4% (vol/vol) formaldehyde and sectioned to 3‐µm‐thick sections. The heart sections were stained with Masson's trichrome to assess the formation of fibrotic tissue after infarction according to the manufacturer's instructions. Images were captured under inverted fluorescence microscope (Leica) or captured on a Leica SP8 confocal microscope. The size of the blue‐stained fibrotic area and the left ventricle wall thickness were measured by ImageJ software. Three heart samples per group were used for the analysis.

### Statistical Analysis

All experiments were performed independently at least three times, and the results were presented as mean ± SEM. Comparisons between two groups were performed using two tailed unpaired Student's *t*‐test. Comparisons among more than two groups were performed using one‐way ANOVA followed by Turkey's post hoc test. Quantitative data plotting and statistical calculations were performed in GraphPad Prism version 8.0 software. *p* < 0.05 was considered a statistically significant difference. The significance level is indicated as *p < 0.05, ^**^ *p* < 0.01, or ^***^ *p* < 0.001.

## Conflict of Interest

The authors declare no conflict of interest.

## Author Contributions

Y.L., Y.Z., and T.M. contributed equally to this work. W.L. and Z.L. supervised and conceived the project. Y.L. carried out the experiments and acquired the data. Y.L., W.L., and Y.H. analyzed and interpreted the data. Y.Z., T.M., and Z.W. contributed to 3D bioprinting and cardiac patch engineering. T.M. and H.C. carried out the animal experiments and the data analyses. W.J. contributed to hESCs culture. J.W. and Z.Z. provided technical expertise in immunohistochemical experiments. Y.L. drafted the manuscript. Y.Z. and T.M. contributed to data interpretation and manuscript preparation. W.L., Y.H., and Z.L. revised the manuscript and all the authors reviewed the manuscript.

## Supporting information

Supporting Information

Supplemental Movie 1

Supplemental Movie 2

Supplemental Movie 3

Supplemental Movie 4

Supplemental Movie 5

Supplemental Movie 6

Supplemental Movie 7

Supplemental Movie 8

Supplemental Movie 9

## Data Availability

The data that support the findings of this study are available from the corresponding author upon reasonable request.
