# Supplementary material for: hESCs‐Derived Early Vascular Cell Spheroids for Cardiac Tissue Vascular Engineering and Myocardial Infarction Treatment
Source: Adv Sci (Weinh). 2022 Jan 29;9(9):2104299. doi: 10.1002/advs.202104299 (PMC8948571; doi:10.1002/advs.202104299)
Supplement: Supplementary file 1 — Supporting Information [file ADVS-9-2104299-s008.pdf]

ADVANCED SCIENCE
Open Access

Supporting Information

for *Adv. Sci.*, DOI 10.1002/advs.202104299

hESCs-Derived Early Vascular Cell Spheroids for Cardiac Tissue Vascular Engineering and Myocardial Infarction Treatment

Yang Liu, Yifan Zhang, Tianxiao Mei, Hao Cao, Yihui Hu, Wenwen Jia, Jing Wang, Ziliang Zhang, Zhan Wang, Wenjun Le and Zhongmin Liu**

ADVANCED SCIENCE
Open Access

Supporting Information

for *Adv. Sci.*, DOI: 10.1002/advs.202104299

hESCs-Derived Early Vascular Cell Spheroids for Engineering Vascularized Cardiac Tissues and Myocardial Infarction Treatment

Yang Liu[1,#], Yifan Zhang[1,#], Tianxiao Mei[1,2,#], Hao Cao[3], Yihui Hu[1], Wenwen Jia[2], Jing Wang[1], Ziliang Zhang[1], Zhan Wang[4], Wenjun Le[1,*], Zhongmin Liu[1,2,3,*]

Supporting Information

hESCs-Derived Early Vascular Cell Spheroids for Engineering Vascularized Cardiac Tissues and Myocardial Infarction Treatment

Yang Liu[1,#], Yifan Zhang[1,#], Tianxiao Mei[1,2,#], Hao Cao[3], Yihui Hu[1], Wenwen Jia[2], Jing Wang[1], Ziliang Zhang[1], Zhan Wang[4], Wenjun Le[1,*], Zhongmin Liu[1,2,3,*]

[1] Institute for Regenerative Medicine, Shanghai East Hospital, Frontier Science Center for Stem Cell Research, School of Medicine, Tongji University, Shanghai 200092, China
[2] National Stem Cell Translational Resource Center, Shanghai East Hospital, School of Life Sciences and Technology, Tongji University, Shanghai, 200092, China
[3] Department of Cardiovascular Surgery, Shanghai East Hospital, Tongji University School of Medicine, Shanghai 200120, China
[4] Department of Internal Medicine, Section on Molecular Medicine, Wake Forest School of Medicine, Medical Center Blvd, Winston-Salem, NC 27157, USA
[#] These authors contributed equally to this work.
***** Correspondence: E-mail: liu.zhongmin@tongji.edu.cn, wenjunle@tongji.edu.cn

Supplementary Tables

Table S1: Primer sequences used for qPCR analysis

Genes	Sequence of forward primer	Sequence of reverse primer
cTnT	TTCACCAAAGATCTGCTCCTCGCT	TTATTACTGGTGTGGAGTGGGTGTGG
PECAM1	GACGTGCTGTTTTACAACATCTC	CCTCACGATCCCACCTTGG
VE-cadherin	GACCGGGAGAATATCTCAGAGT	CATTGAACAACCGATGCGTGA
Brachyury (T)	AGTGAGCTGACAGGGTTGCT	CCATTGGGAGTACCCAGGTT
KDR	ACTTTGGAAGACAGAACCAAATTATCTC	TGGGCACCATTCCACCA
CD34	AAATCCTCTTCCTCTGAGGCTGGA	AAGAGGCAGCTGGTGATAAGGGTT
POU5F1	GCAATTTGCCAAGCTCCTGAA	AAGCTAAGCTGCAGAGCCTCAAAG
NANOG	CAACTGGCCGAAGAATAGCAATG	TGGTTGCTCCAGGTTGAATTGTT
GAPDH	TGCACCACCAACTGCTTAGC	GGCATGGACTGTGGTCATGAG

Supplementary Figures

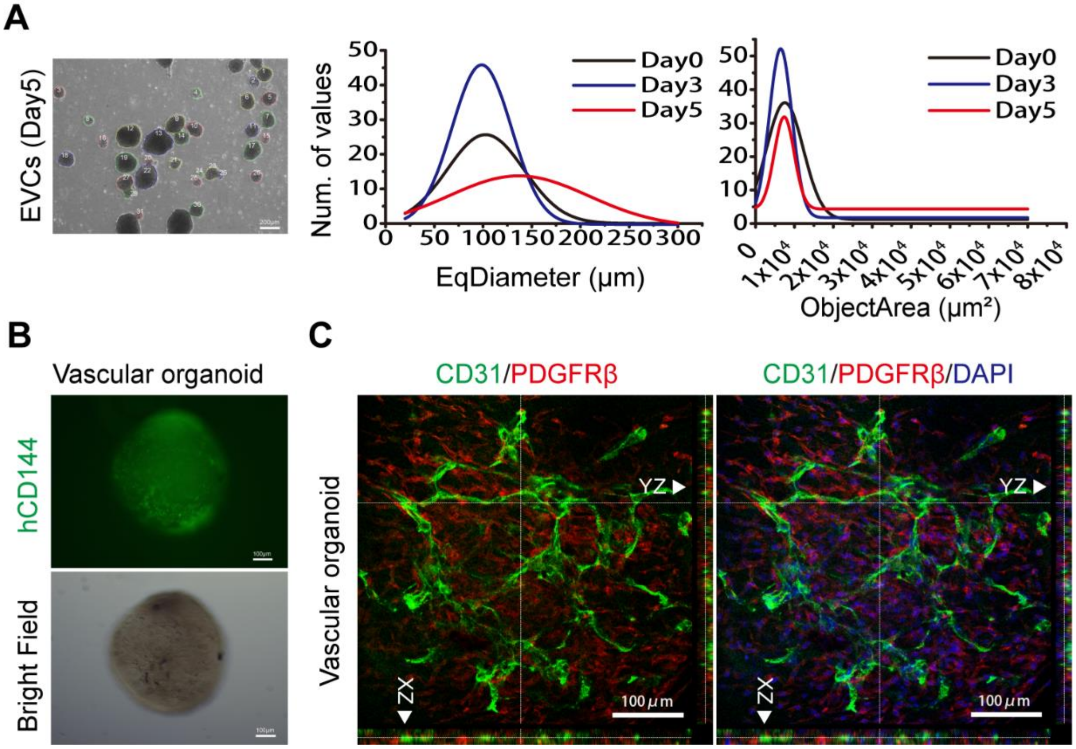

Figure S1. Differentiation and fabrication of human vascular organoids from hESCs. (**A**) Quantitative analysis of EB size at Day0, Day3 and Day5. Representative analytic results of EVCs at Day5 using NIS-Elements software were shown on the left. Scale bars, 100 μm. Frequency distribution of equivalent diameter and objective area were analyzed and fitted by nonlinear regression with Gaussian equation using OriginPro 9.0 software. (**B**) Vital staining of human CD144-FITC in vascular organoid constructed from H9 hESCs. Scale bars, 100 μm. (**C**) Immunofluorescence staining of CD31 and PDGFRβ in vascular organoid showed endothelial cells and pericytes are in close interconnection. Scale bars, 100 μm. EB indicates embryoid body.

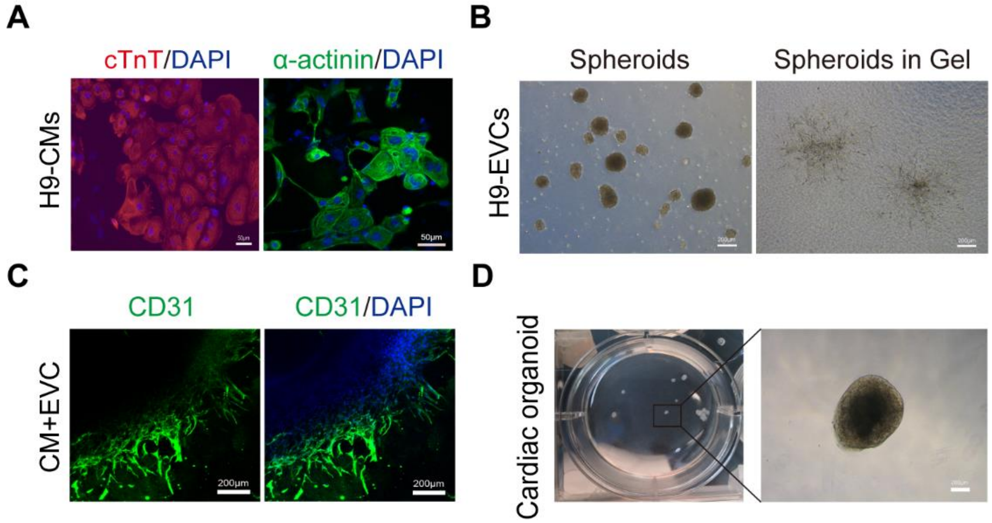

Figure S2. Fabrication of cardiac microtissues by 3D co-culture of hESCs-derived vascular and cardiac cells. (**A**) Immunofluorescence staining of cTnT and α-actinin in CMs after lactate-based purification of cardiac differentiation from H9. Scale bars, 50 µm. (**B**) Bright field images of aggregated spheroids with dissociated EVC single cells and sprouting detection after embedding in ECM hydrogel. Scale bars, 200 µm. (**C**) IF staining of CD31 in spheroids of CMs and EVCs embedded in Collagen I-Matrigel matrix showed sprouting of endothelial cells. Scale bars, 200 µm. (**D**) Bright field images of representative cardiac organoids with round and fully encapsulated morphology. Scale bars, 200 µm. CM, cardiomyocytes; EVC, early vascular cell.

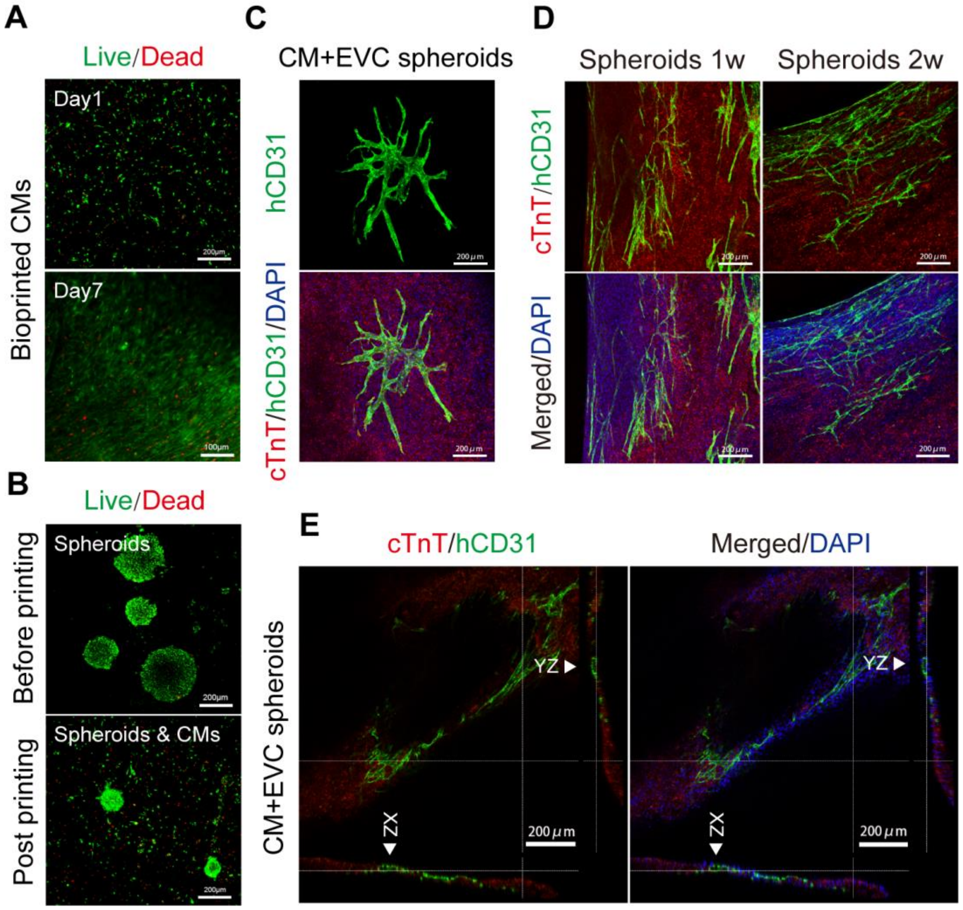

Figure S3. Detection of human endothelial cells and vascular networks in 3D bioprinted cardiac patch. (**A**) AO-PI staining of bioprinted neonatal rat CMs in cardiac patches post-printing Day1 and Day7. Scale bars indicate 200 μm and 100 μm, respectively. (**B**) AO-PI staining of EVC spheroids before and after the bioprinting process. Scale bars, 200 μm. (**C**) Immunofluorescence staining of cTnT and human CD31 in cardiac patches of CMs and EVC spheroids showed spider-like endothelial cell sprouting. Scale bars, 200 μm. (**D**) IF staining of cTnT and human CD31 in cardiac patches with EVC spheroids and cultivated for 1 week and 2 weeks showed connected endothelial cells. Scale bars, 200 μm. (**E**) Orthogonal projection of IF staining in 3D bioprinted cardiac patch with CMs and EVC spheroids showed vascular lumens enveloped by $hCD31^+$ endothelial cells have generated. Scale bars, 200 μm. CM, cardiomyocytes; EVC, early vascular cell.

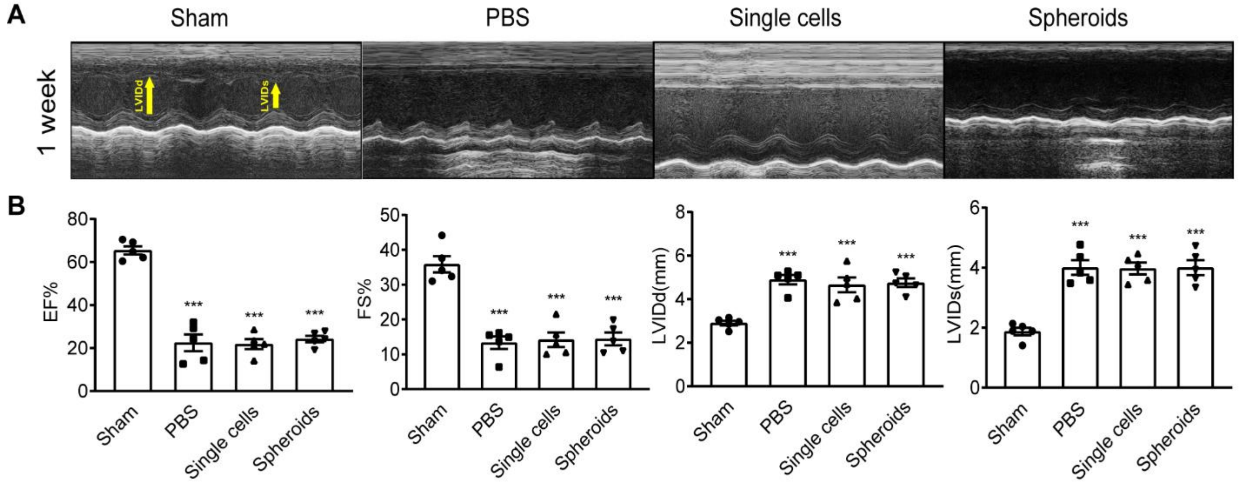

Figure S4. Echocardiographic measurements of cardiac functions post-MI at 1 week. (**A**) Representative images of echocardiography after MI for 1 week in different groups (n=5 mice per group). (**B**) Echocardiographic measurements of cardiac functions at 1 week post-MI and cell transplantation (n=5 mice per group). Data were shown as mean ± SEM and analyzed using one-way ANOVA followed by Tukey's post-hoc analysis to show significance compared to sham group. Statistical significance was defined as $P<0.05$ (***$P<0.001$, **$P<0.01$ or *$P<0.05$; ns means no significance). MI, myocardial infarction; EF, ejection fraction; FS, fractional shortening; LVIDd, left ventricular internal dimension-diastolic; LVIDs, left ventricular internal dimension-systolic.

Supplementary Movies

Movie S1: 3D reconstitution of immunofluorescence staining of vascular organoid derived from H9 hESCs. Vascular endothelial cells positive for CD31 were stained as green and cell nucleus were stained as blue.

Movie S2: Day 12 CMs derived from H9 hESCs induced by monolayer-based method show spontaneous beating (10×).

Movie S3: Cell aggregates of CMs co-cultured with EVCs show spontaneous beating after embedding in ECM hydrogel, note the evident sprouting of cell aggregates (10×).

Movie S4: Video from microscope in bright field shows the spontaneous beating of an extracted and encapsulated vascularized cardiac organoid (10×).

Movie S5: Confocal stacks of vascular networks in cardiac tissues. Vascular endothelial cells positive for human CD31 were stained as green and rat CMs were stained as red (10×).

Movie S6: Representative video showing the spontaneous beating of 3D bioprinted cardiac patch with neonatal rat CMs only. Scale bars, 100 μm.

Movie S7: Representative video showing the spontaneous beating of 3D bioprinted cardiac patch with neonatal rat CMs and EVC spheroids. Scale bars, 100 μm.

Movie S8: Calcium imaging analysis shows synchronization contraction of 3D bioprinted cardiac patch with neonatal rat CMs only. Scale bars, 100 μm.

Movie S9: Calcium imaging analysis shows synchronization contraction of 3D bioprinted cardiac patch with neonatal rat CMs and EVC spheroids. Scale bars, 100 μm.
